# Medical Education

**Published:** 1890-09

**Authors:** 


### MEDICAL EDUCATION.

We take pleasure in publishing in this issue of THE JOURNAL "A Plea for Higher Medical Education," by Dr. W. G. Hardman, the leading sentiment of which we most heartily endorse. It must be a source of constant chagrin and mortification to every thinking American physician who has the least tinge of national pride, that none of our medical colleges are recognized by European universities. This lack of recognition is not because we have no excellent medical institutions, but is due to the fact that the few good colleges are legally on a par with a host of inferior ones, whose impecunious facilities must offer all sorts of inducements to attract students and thereby increase their meagre incomes. Nor is it to be expected that any reform will come through such institutions whose very existence makes general medical excellence in the United States impossible. There seems to be no limit to the chartering of medical colleges. Our representatives apparently make no effort to investigate the responsibility, capacity or education of those who make application for such charters. Not many years ago there unexpectedly sprang into existence a so-called medical college in this city the dean of whose faculty was not even a graduate of any school of medicine. Now that institution, short-lived though it was, issued diplomas to a few students, and those diplomas are legally on a footing with those of the best colleges in this country, the holders thereof being entitled to practice medicine or surgery without let or hindrance. It is impossible for the laity to always properly estimate those who claim to be physicians, and it is a deplorable fact that a thoroughly educated, high-toned physician, who is too much of a gentleman to drum up business, must frequently see himself outstripped by a smooth-tongued ignoramus who understands manipulating the public. The people are not educated in this direction, their chief sources of information upon things medical being the quack and patent medicine advertisements in the newspapers and almanacs. Nor can we hope that the newspapers will exclude such advertisements, to say nothing of speaking out boldly against them, so long as they are such a fruitful source of revenue. The cry of class legislation is always raised when there is any attempt to pass laws tending to elevate the general tone of the profession and the newspapers are ever ready to ridicule, to characterize as old fogies, etc., those high-toned physicians who subscribe to the code of ethics and who are unwilling to stoop to secular advertising.

We cannot hope for any sudden or revolutionary changes in the public mind. Real reforms unfold slowly, and the profession as a body can only work diligently and wait patiently for that gradual evolution of sentiment which must occur. Nor are we to be mere passive factors in that evolution, but positive, active elements. Not from the ordinary medical college need we expect much coöperation under existing conditions. Through the members of the profession individually and collectively must those potent agencies become active. But how are we to work? We can demand of those students to whom we act as preceptors and for whose medical careers we become in a great measure responsible, that they obtain better preliminary educations before entering upon the study of medicine. We can send them to those colleges that offer the best facilities for teaching and whose standards for graduation are highest. We can dissuade from attempting to enter the profession men whose preliminary educations are deficient and who have neither the money to obtain nor the capacity to receive scientific medical educations. By these means we can accomplish much, and we owe it to our noble profession and to humanity to break the bonds of indifference, to shatter the shackles of indolence and to put our shoulders to the wheel like men who have the courage of their convictions and who feel the responsibilities with which they are clothed.

The subject should be continually agitated until the laity become fully awakened to their own interests, and then we can obtain laws that will secure a far higher standard of medical education, that will close the doors of those colleges who owe their existence to a desire for notoriety and pecuniary benefit on the part of their faculties, and whose constant tendency is to lower instead of to raise the standard of medical education.

### ITEMS.

DR. VIRGIL O. HARDON is spending his summer vacation in New England.

THE ATLANTA SOCIETY OF MEDICINE.—The first meeting of the Society after the summer vacation will be held on Tuesday, the 2d inst. A full attendance is very much desired, as the question of a new hall will be decided. The President, Dr. Nicolson, will submit a report thereon.

INTERNATIONAL MEDICAL CONGRESS.—The 10th International Medical Congress, held in Berlin, August, 1890, was the largest and most notable gathering of medical men ever assembled. The meeting seems to have been a great success, both from social and scientific standpoints.

Professor Virchow's address of welcome, was replete with high and philanthropic sentiments, among them the following: "They one and all gloried in the fact that the great personal sacrifices often made by doctors were frequently not attended with any pecuniary compensation, and the medical men of the whole world were not gathered in such immense meetings to gain personal advantages, to improve their social position, to receive a higher rate of pay and less work, but only to improve in knowledge, to render themselves strong in the power to cure, to render still greater services to their fellow creatures than before."

Representatives were there from almost every civilized country, America being represented by several hundred. The next Congress will be held in Rome.

### TRI-STATE MEDICAL ASSOCIATION.

The next meeting will be held in Turner Hall, Chattanooga, Tenn., August 15 and 16. The outlook for a large attendance is good. The hall is well adapted for the meeting, having a large room adjoining for exhibits. The committee has secured rates on railroads of one and one-third fare on the certificate plan. Georgia should send up a good delegation to this association as it is a society worthy of support. Those intending to read papers should send letters to Dr. Frank Trester Smith, Chattanooga, Tenn., before September 15.

CROWDS OF DOCTORS EVERYWHERE.—Those who are interested in reading the names of passengers across the Atlantic, must have been struck by the excessive number of medical men on the lists of our ocean steamers. But in spite of this exodus, all our inland watering-places, all our mountain and sea-shore hotels, all summer resorts where more than ten families may be expected to flock together, have retained their undue proportion of physicians. It will soon happen that the medical fraternity will have only doctors and their families for patients. Professional charity will then have reached its high-water mark, and "doctor's bills" will be but a memory of the past.—*New York Medical Record*.

### THE EXCLUSION OF LIGHT NOT BENEFICIAL IN THE AFTER-TREATMENT OF CATARACT OPERATIONS.

Dr. Julian J. Chisholm, of Baltimore, writing on the above subject in the New York *Medical Record* of August 2, 1890, gives a brief résumé of his work and the results obtained under this plan of treatment during the last four years. He simply uses a strip of "salicylated soft silk, isinglass plaster, one and a half inch long and one inch wide, placed over the closed eyelids." The plaster does not cover completely the canthi, thus allowing the secretions to escape. The opposite eye is not covered The patients are not confined to bed, and there is no attempt to secure more exclusion of light than that afforded by an ordinary window shade. The doctor claims much better results in all classes of cataract operations than he obtained under the old compress and bandage dressings. The recovery is more rapid and without that watery, irritable, congested conditions of the eye found upon the removal of the old dressings.

The American Rhinological Association will hold its eighth annual session at Louisville, Ky., October 6th, 7th and 8th.

All leading subjects relating to nasal and naso-pharyngeal diseases will be opened for discussion by a leading fellow of the association. The medical profession is cordially invited to attend.

The secretary, Dr. R. S. Knode, Omaha, Nebraska, will furnish any information to physicians desiring to become members.

SECRECY IN LYING-IN HOSPITALS.—Last week we expressed the hope of seeing institutions established here for enabling women pregnant out of wedlock to be assured of decent support and secrecy until they were relieved of their embarrassment by the birth of a full-time child and recovery from the disabilities of the lying-in period. This we said in the interest of the restriction of criminal abortion. In the course of an essay on the proper measures for remedying the depopulation of France, an abstract of which appears in a recent number of the *Union Medicale*, M. Lagneau advocates the establishment of such institutions, and alludes to their existence in Vienna. The officers and employees are sworn to secrecy, and there a woman may be delivered and leave her child behind her when she is ready to be discharged, without her identity being made known.—*The New York Medical Journal, August, 1890.*

NITRATE OF COCAINE IN THE URINARY PASSAGES.—Dr. Lavaux thinks the nitrate of cocaine should replace the hydrochlorate of the drug for genito-urinary employment, where nitrate of silver is to be used. In the Journal de med. of March 23, 1890, he relates some experiences with the nitrate in connection with somewhat strong nitrate-of-silver injections. The following formula is given as one recently employed in gonorrhœa without the production of pain:

℞. Distilled water.................. 50 grammes;
Nitrate of cocaine.............. 1 gramme;
Nitrate of silver................ 1 gramme;

The canal is to be washed out with a four-per-cent. boric solution before and after injection.

The nitrate of cocaine is readily prepared by pouring a solution of the hydrochlorate of cocaine into a solution of nitrate of silver. By double decomposition a precipitate of chloride of silver, wholly insoluble, falls down, and the nitrate of cocaine remains in solution.—*Journal of Cutaneous and Genito-Urinary Diseases, July, 1890.*

THE ADMINISTRATION OF CHLOROFORM BY GASLIGHT.—Considerable attention has been given of late to the chemical composition of the compounds formed by chloroform vapor, air, and the products of the decomposition of coal gas. It appears from the investigations of several chemists (Stobwasser, von Iterson, Zweifel, and others) that chloroform vapor may be decomposed by gas flame and give rise to a compound of carbon and chlorine which is very irritating to the respiratory organs. This substance, along with others, forms a vapor in the neighborhood of the gas jet or of the petroleum flame. The operators sometimes experience pains in the head, nausea, and dizziness, while the subjects of operation suffer afterward from dyspnœa, cough and lacrymation. Asphyxia in the course of the anæsthesia may develop at any moment and be followed by pulmonary complications. Kunckel's experiments (*Bulletin Medical*) show that the chloroform is decomposed into hydrochloric acid, and he believes that it is that which does the mischief. He thinks that the effect might be counter-acted by inhalation through linen soaked in an alkaline solution.—*The New York Medical Journal, August, 1890.*

Mr. Cornelius Vanderbilt, in conjunction with his mother, Mrs. Wm. H. Vanderbilt, has given $250,000 for the erection and maintenance of a building on East Forty-Second Street, to be known as St. Bartholomew's Mission, which is designed for the health and comfort, as well as the morals, of the tenement population in the neighborhood. It will be constructed of white brick with dark red terra-cotta trimmings, and will be six stories high, with a depth of one hundred feet and width of seventy-five feet. In the basement there will be a large swimming bath for the use, at different hours, of both sexes, with a heating apparatus for cold weather. On this floor will also be a kitchen and dining-room and a work-room for the temporary employment of people out of work. The main floor will be occupied by the chapel and a large hall for mission services, which will be two stories in height. The third floor will be devoted to rooms for the Sunday-school and for mothers' meetings and sewing classes; and the fourth to a library, reading-room, boys' club, and assembly and class rooms. The fifth room will be fitted up as a fully equipped gymnasium, to be open at different hours to both men and women, and on the upper floor will be the offices and living rooms of those engaged in the administration of the establishment. The whole is to be under the direction of the rector of St. Bartholomew's church.—*The Boston Medical and Surgica, Journal, July, 1890.*

TREATMENT OF BUBO.—According to Dr. Cordier, of Lyon, the procedure which has given him the best results in the treatment of bubo is as follows: As soon as œdema of the skin shows the presence of pus, he make a puncture with a straight bistoury. There is discharged along with the blood some streaks of pus. No pressure is made, but the cavity is injected with about a cubic centimetre of a 1-to-50 solution of nitrate of silver. Without paying any attention to whether the solution flows out again or not, a dressing is made with iodoform and a spica bandage. When this slight operation is made early enough, no purulent discharge follows, and the bubo disappears rapidly in leaving behind a sort of indurated nodule. When the puncture is done in a bubo much more advanced and in full suppuration, the purulent collection must be carefully washed out. A first injection of the solution mentioned is made which is destined to cleanse the parts, and the second injection is left in ; a decided imflammatory reaction is established and quite an abundant suppuration occurs and lasts for three or four days and then becomes serious. Healing takes place rapidly, and the opening closes without leaving a cicatrix. Dr. Cordier has had the same results, whether the bubo was chancrous or simply inflammatory.—*Journal of Cutaneous and Genito-Urinary Diseases, July, 1890.*

PARTIAL RESECTION OF THE DISEASED OVARY AND TUBE.—Dr. Martin, of Berlin, has systematically practiced the partial removal of ovaries not entirely diseased. In some cases he has also resected part of the tube, and made, by suture of the mucosa to the serous coat, a new ostium. This proceeding has been termed "salpingostomy" by Dr. Skutsch, of Jena, and has also been advocated by Wallace and Schröder. Dr. Martin, as the result of his experience, published in Volkmann's *Klinischer Vorträge*, came to the following conclusions : Patients recover perfectly after partial removal of ovaries for localized chronic inflammatory changes, hydrops folliculi and oöphoritis. Recovery is also complete, in most cases, after the resection of obstructed and otherwise diseased tubes. The after-histories of seventeen patients operated upon by Dr. Martin prove that women with resected ovaries and tubes are not more exposed than other women to further disease of the parts left behind. Menstruation continued in all the cases, and some conceived. Dr. Martin notes that in 1864 Sir Spencer Wells emptied some dropsical follicles in one ovary of a young girl, having just removed its fellow. The girl afterwards married, and had children. The first resection of the ovary, performed intentionally, was undertaken by Schröder in 1884. A dermoid cyst occupied part of an ovary (the fellow in this case also formed a tumor and was removed), it was cut out, and the wound in the ovary was sewn up. The patient appears, according to a note in Holmeier's *Grundriss der gynäkologisch. Operat.*, to have conceived after the publication of Schröder's own report of her case.—*British Medical Journal, August, 1890.*

INDUCTION OF PREMATURE LABOR.—In these days when abdominal section is so freely practiced obstetricians of authority tend to throw discredit upon the induction of premature labor and craniotomy, and so advocate Cæsarean section. Professor Ahlfeld, however, has lately issued an important monograph on 118 cases of induction of labor, performed by himself and his assistants between 1871 and 1890, at Leipzig, Giessen and Marburg. In 111 of these cases labor was induced on account of deformed pelvis; in the remaining seven it was undertaken because of some general illness. Only one mother appears to have died from the direct results of the proceeding in question; she succumbed to damage of the soft parts caused by the passing of a large head through an atypical pelvis. Four mothers died from diseases which either followed accidentally or were those for which labor had been induced (convulsions, tuberculosis, heart disease); seventy-five mothers recovered without rise of temperature. One hundred and twenty-one children were thus delivered (there being three cases of twins in the 118 labors). Of these nineteen were still-born, eighteen died in the course of the first day, nine died before the mother was discharged, and seventy-five left in the hospital, etc., alive. Out of ninety-nine carefully registered contracted pelvis cases (including two twin labors) eighty-six children were born alive, fifteen still-born, and sixty-one survived. On the other hand, in six cases of Cæsarean section performed in Professor Ahlfeld's wards, two mothers and one child died. Space forbids any notice of the author's statistics as to the extent of contraction in the pelves.

The monograph* deserves close study. Professor Ahlfeld insists that the introduction of a flexible bougie into the uterus without rupture of the membranes is by far the best method, but the pains are apt to be slow, so that accessory means are sometimes required. There can be no doubt that the majority of practical obstetricians are more likely to attain good results by inducing labor than by performing Cæsarean section or Porro's operation.—*British Medical Journal, August, 1890.*

OBSERVATIONS ON SOMNAL.—Somnal is a compound or mixture of chloral, urethan, and alcoholic ethyl. It appears as a colorless fluid which, on cooling, takes the form of needle-shaped crystals. It has a bitter taste. It has been tried with good results by Dr. Constantin Paul in articular rheumatism, and also by Dr. Guttmann, who found that it produced a pleasant sleep lasting from six to eight hours, without any bad effects. (*The London Medical Recorder.*) In some cases, however, it fails in its action. Dr. Zagorski, of Warsaw, prefers it to all other hypnotics for the rapidity of its action, the duration and intensity of the sleep, and for the absence of any effects upon the bowels, the heart, the lungs, and the general temperature. The dose recommended is about thirty grains in a little distilled water and syrup of orange. It is deliquescent and soluble in water, and in one-third of its weight in alcohol. Others have treated it as a mixture of uncertain composition, of a bad, bitter taste, and less powerful than chloral. Lutze found it only efficacious in thirty per cent. of his cases, and only useful in fifteen per cent.; and in eight cases of great excitement it was found to act more quickly than chloral. Drs. Venanzio and Sighicelli, of Milan, have tried somnal in eighty patients in doses of from two to four grammes. In some few cases they went as high as six grammes. The general results observed by these physicians were as follows: Somnal produces no gastric disturbance and neither affects the appetite nor the digestion. Sometimes the pulse becomes more frequent and weaker a little while after taking the drug, but this effect soon passes away. Somnal has no action on the respira-

*Centralblatt fuer Gynakologie, July 26th, 1890.

tion, but sometimes produces a redness of the face dependent upon paralysis of the vessels, which is easily increased by the addition of a small quantity of alcohol. In doses of thirty grains somnal may be used for slight restlessness, but in cases of deep insomnia, dependent on cerebral excitement, it is necessary to give as much as sixty grains, which generally produces a sleep of from five to six hours. It rarely happens that sleep comes on in half an hour ; in general it takes about three hours, sometimes even as much as four or five, till the desired effect is obtained. In cases accompanied with mental depression, somnal acts better than where there is much excitement. On the whole, they consider that somnal is a useful hypnotic, from the duration and quality of the sleep which it induces, from its having no bad effects, and also from its small cost. In its therapeutic effects it has a resemblance to its two principal components, chloral and ether.—*Medical Record*, *August*, *1890*.

SCHMITZ. "Is diabetes transmissible ? "—*Berlin*, *klin. Woch.*, May 19, 1890.

More than 15 years before the discovery of the bacillus, Dr. Hermann Weber (London) drew attention to the transmission of tubercle in married couples. Up to 1880, 600 diabetic patients passed through Schmitz's hands, and of these, eight cases were those of both man and wife affected with the disease, there being no reason (neither of inheritance nor fondness of sweets) why the disease should attack each in succession. This was suggestive, and when in 1890 there were 26 such cases out of 2,320 patients (diabetic), the obvious suggestion was that diabetes is transmissible. To put it in Schmitz's own words :—" Persons known to be thoroughly healthy, mostly all married with but few exceptions, and, for the most part females, after they had for a considerable time nursed, slept in the same chamber, and been very intimate with, and frequently kissed a diabetic patient, themselves became suddenly diabetic. Not in one single case was the disease inherited ; and also, not in a single case was the last affected connected in the slightest degree of kinship with the first. There was absolutely nothing, apart from transmission, to fix upon as a cause of the diabetes, as the patients had in particular never eaten much sugar, nor suffered from arthritis, etc."

The number of cases, over 1 per cent., almost puts chance out of the question, and the more so when almost all the 26 occurred under similar circumstances.

Like Weber with tubercle, and suggesting evidently some similar explanation, Schmitz concludes that, in cases of exceptional long-continued intimacy, diabetes is transmissible. He regrets that he is not in a position to establish his theory by experiment on the lower animals with diabetic blood and the contents of the stomach and intestines of diabetics. Possibly, he suggests, only one form of diabetes is transmissible, and not all forms.

He ends his paper with seven cases, and they certainly give his theory remarkable probability. [As regards the female being far more liable to infection from proximity than the male, one may suggest that, as in cattle, a male of impure breed will contaminate the future offspring of the cow; so in diabetes, the constitution of the husband might so influence that of the wife that the diabetic tendency is also produced.]—*Medical Chronicle August, 1890.*

HARE (F. W. E.). "The true value of quinine in continued fevers."—*Australasian Medical Gazette, April, 1890.*

As the result of a most extensive trial of the drug in continued fevers, the author concludes that quinine is a very valuable cardiac stimulant. It slows and at the same time strengthens the heart's action, and in uncomplicated cases completely holds in check the tendency to cardiac failure.

Dr. Hare gives from three to five grains or more every three hours, and thinks that the drug should be prescribed in this manner whenever the pulse attains a frequency of 120 per minute. The slowing of the pulse is late in appearing, twenty-four hours usually elapsing before it is distinctly manifest. On the other hand, the effect once obtained is long continued; for if the drug be withheld, the pulse does not attain its former rage of frequency for two or three days.—*Medical Chronicle, July, 1890.*

### THE NECESSARY PEROXIDE OF HYDROGEN.

BY ROBT. T. MORRIS, M. D., OF NEW YORK.

Stop suppuration! That is the duty that is imposed upon us when we fail to prevent suppuration.

As the ferret hunts the rat, so does peroxide of hydrogen follow pus to its narrowest hiding place, and the pyogenic and other microörganisms are as dead as the rat that the ferret catches, when the peroxide is through with them. Peroxide of hydrogen $H_2O_2$ in the strong 15-volume solution is almost as harmless as water, and yet, according to the testimony of Gifford, it kills anthrax spores in a few minutes.

For preventing suppuration we have bichloride of mercury, hydronaphthol, carbolic acid, and many other antiseptics, but for stopping it abruptly and for sterilizing a suppurating wound we have only one antiseptic that is generally efficient so far as I know, and that is the strong peroxide of hydrogen. Therefore I have qualified it, not as "good," not as "useful," but as necessary."

In abscess of the brain, where we could not thoroughly wash the pus out of tortuous canals without injuring the tissues, the $H_2O_2$, injected at a superficial point, will follow the pus, and throw it out, too, in a foaming mixture. It is best to inject a small quantity, wait until foaming ceases, and repeat injections until the last one fails to bubble. Then we know that the pus cavity is chemically clean, as far as live microbes are concerned.

In appendicitis, we can open the abscess, inject peroxide of hydrogen, and so thoroughly sterilize the pus cavity that we need not fear infection of the general peritoneal cavity if we wish to separate intestinal adhesions and remove the appendix vermiformis. Many a patient, who is now dead, could have been saved if peroxide of hydrogen had been thus used when he had appendicitis.

The single means at our disposal allows us to open the most extensive psoas abscess without dread of septic infection following.

In some cases of purulent conjunctivitis, we can build a little [illegible]f wax about the eye, destroy all pus with peroxide of hy–

drogen and cut the suppuration short. Give the patient ether if the $H_2O_2$ causes to much smarting. It is only in the eye, in the nose and in the urethra that peroxide of hydrogen will need to be preceded by cocaine (or ether) for the purpose of quieting the smarting, for it is elsewhere almost as bland as water.

It is possible to open a large abscess of the breast, wash it out with $H_2O_2$, and have recovery ensue under one antiseptic dressing, without the formation of another drop of pus.

Where cellular tissues are breaking down, and in old sinuses, we are obliged to make repeated applications of the $H_2O_2$ for many days, and in such cases I usually follow it with balsam of Peru, for balsam of Peru, either in fluid form or used with sterilized oakum, is a most prompt encourager of granulation.

If we apply $H_2O_2$ on a probang to diphtheritic membranes at intervals of a few moments, they swell up like whipped cream and come away easily, leaving a clean surface. The fluid can be snuffed up into the nose and will render a fetid ozæna odorless.

It is unnecessary for me to speak of further indications for its use, because wherever there is pus we should use peroxide of hydrogen. We are all familiar with the old law, "*Ubi pus, ibi evacua*," and I would change it to read, "*Ubi pus, ibi evacua, ibi hydrogenum peroxidum infunde.*" That is the rule. The exceptions which prove the rule are easily appreciated when we have them to deal with.

Peroxide of hydrogen is an unstable compound, and becomes weaker as oxygen is given off, but Marchand's 15-volume solution will retain active germicidal powers for many months, if kept tightly corked in a cold place. The price of this manufacturer's preparation is about 75 cents per lb., and it can be obtained from any large drug house in this country. When using the $H_2O_2$ it should not be allowed to come into contact with metals if we wish to preserve its strength, as oxygen is then given off too rapidly.

$H_2O_2$ must be used with caution about the hair if the color of the hair is a matter of importance to the patient, for this drug, under an alias, is the golden hair bleach of the *nymph's dispare*, and a dark-haired man with a canary-colored moustache is a stirring object.—*Journal of American Medical Association, August 9, 1890.*

DOUBLE PERSONALITY.

In some of the most difficult problems of life that are at least in part physiological, the rare abnormalities of Nature give a leading clue to the laboratories of experimental physiology, which is by no means to be neglected, though it may be hard enough to explain or thoroughly to follow out. An instance of this occurs in a curious bit of personal history which came lately under the notice of Dr. Proust, the distinguished physician and Professor of Hygiene at the Hotel Dieu, in Paris. On this he has read lately a remarkable paper to the Academy of Moral and Political Science in Paris. It has filtered through several Parisian correspondents into the English press, and has excited some comment. It is one of those instances of double personality which are so rare to English eyes, in which both philosophers and physiologists can hardly fail to be interested, for it offers problems to both which are not easy to solve, and indeed it would be a feather in the cap of any one who could provide a complete and satisfactory solution.

The story* plainly told is this: Emile X., aged 33, is a barrister in Paris, a well-educated man, successful in his classical studies as a boy, who spent a short time as medical student before he turned to law. His father had been an eccentric man and a heavy drinker; his younger brother was mentally deficient. He was himself always very impressionable, and sometimes a loud and sudden noise was enough to send him into what was considered a hypnotic sleep. Even in the law courts on one occasion when conducting a case, the judge fixed his eyes upon him, and thereupon he stopped speaking and fell into this abnormal state. His friends, who knew his peculiarity, roused him at once, and then he went on with his speech. Again, when he was at a *café* near the bank, he looked at himself in the mirror and fell asleep again. His neighbors were surprised, and did not understand it,

* *Revue de l' Hypnotisme*, Mars, 1890, *p.* 267.

and thereupon took him to the Charité, where he was awakened. The strangest thing about him was his occasional loss of memory for considerable lengths of time, and his change of personality during these times. He was wide enough awake throughout, but whilst he was in this condition he could remember nothing at all of his past life. He was active in every way, walking, riding, travelling by train, or making purchases in the shops. And when he passed back from this secondary life to his normal state, it was by a process of awakening, and he had no recollection at all of what he had been doing. He had two memories, in point of fact, which alternated—the first belonging to his normal life, the second to his abnormal. On September 23d, 1888, he had a quarrel with his stepfather in Paris, which reduced him to his second self, and this he did not cast off for three weeks, when he found himself in a village a hundred miles from Paris. He could not tell how he had gotten there, or what he had done ; he could find out from questioning his neighbors that he had paid a visit to the priest of the village who had thought his conduct odd, and previously to that he had stayed with an uncle who was a bishop, and in whose house he had broken his furniture and torn up his letters, and had even had a sentence passed against him in a police court for some misdemeanor. During these three weeks he had spent about £20, but where it had gone he did not know.

Again, on May 11th, 1888, he breakfasted as usual in Paris, and found himself some three days after at Troyes, with a similar complete blank in his memory. He noticed that his overcoat was gone, and his purse also, with £9 in it.

Modern experimental psychology has paid a good deal of attention to a few such cases as this, and has been able to advance our knowledge of them considerably and in a useful fashion. Since Azam, of Bordeaux, first published, more than a quarter of a century ago, the outlines of the celebrated case of Félida X., whom he had followed now through thirty-three years of her truly chequered life, we have learnt to appreciate more thoroughly the reality and the meaning of double personality in a few rare cases, such as those of Louis V. and Lucile under MM. Bourru and Burot, of Mdlle. R. L. under Dr. Dufay, as well as those under Verriest and Professor Charles Richet. And in these later years the study of the memory by Professor Ribot and others has revived the interest in the well-known fact that there may be separate memories conjoined with some separation of character and physical conditions; that most somnambulists, for example, have separate memories in their somnambulic and in their normal states. In an attack of somnambulism an unfinished piece of work is sometimes taken up just where it has been left in previous somnambulism, but had never been known of between whiles. When such natural somnambulists are put into the state of *somnambulisme provoque* or hypnotism, it is found that as a rule, the somnambulic memory is recalled, and its ideas and impulses take the place entirely, for the time, of the normal character and normal memory. So, too, on a larger scale, after these longer somnambulisms, or spells of second personality, that may last for days or weeks or even years, the memory of them is generally lost after the return to the primary state, but even then it can be revived in hypnotism. In the case which Professor Proust has related, after Emile X. had spent three weeks of travel and visiting in his second personality, and awakened with no memory of what had happened, Professor Proust goes on to tell us how he was then hypnotized and could, so long as he was hypnotized, recollect his wanderings, his conduct in the Episcopal palace, his losses of money at the card table, and the misdemeanor for which judgment had been given against him. In the same way, after he had been to Troyes, there was no memory of how or why he had gone till he was hypnotized, and then he could tell the whole tale of his journey. This effect of hypnotism was very lucky for him, for it enabled him to describe exactly what he had done with his overcoat and his purse with £9 in it. His friends made a note on this point of what he said in the hypnotic state. After awakening, he was surprised to read his own account, but he believed his hypnotic self, although his normal memory was just as blank as before, and wrote to the hotel at Troyes, telling them where he had left his coat and purse. He was still more surprised when in a couple of days he received from the hotel keeper the coat, found where he had described, and the purse also, with the money in it. The law, too, in a way that is worth notice, acting perhaps on the important precedent in Dr. Dufay's case, of excusing one personality for the criminal actions of the other, revoked its previous judgment against him for the petty misdemeanor of his second personality.—*The British Medical Journal.*

PONFICK (E.). "Experimental contributions to the pathology of the liver." *Virchow's Archive* (Bd. CXVIII., Hft. 2, and Bd. CXIX., p. 193) and *Centralblatt für klinische Medicin*, No. 19, 1890.

In these papers the author gives the results of a very large number of experiments on rabbits with regard to the partial or total extirpation of the liver. One-fourth, one-half, and even three-fourths of the organ were in some cases removed without any bad after-effects. Congestion of the spleen and the walls of the stomach and small intestines was frequently observed, but these changes were not constant. The circulatory disturbances in course of time disappeared. After removal of a portion of the liver, compensation takes place in the liver itself. After the removal of a fourth or one-half of the liver, the remaining lobes present, in a few days even, a totally altered appearance. They are distinctly enlarged. The surface of the liver becomes strongly arched; the appearance similar to that of the spleen, and the consistency very soft. The organ continues to increase in size until, in course of time, complete compensation occurs. A fourth part of the liver is able to increase in size threefold. It possesses the power of so far restoring the mutilated organ that the liver may attain four-fifths of its original size, or may even completely attain its former volume.—*Medical Chronicle, July, 1890.*

ALTHOUGH aristol has been in the hands of the profession such a comparatively short time, an extensive series of publications of experience of its use has appeared, which for the most part quite confirms the very favorable results recorded by Eichoff*

*"Uber die dermat-therap. Wirksamkeit einer neuen jodverbindung das aristol." - *Monaths. f. prakt. Dermat*, X., No. 2.

(abstract in *Medical Chronicle*, March, 1890) of its use. Dr. Brocq reports a remarkable case which he presented to the Société Médicale des Hôpitaux—a patient suffering from extensive superficial epithelioma of the face, extending from the level of the mouth to the orbit, the lower eyelid being completely destroyed. The healing action in this case was at once evident, and in five or six days the wound was becoming covered with a firm cicatrix, and at the time the patient was presented to the Society, after use of the remedy for three weeks, only very small wounds remained. Dr. Brocq has also had most gratifying results in other cases in which he had used the drug, especially ulcers of the legs and ulcerated gummata.

Dr. Gaudin reports cases of psoriasis, ulcers, eczema and chancre, in which he has used the drug with most excellent results; in one case of ulcer of the leg, in which he made comparative experiments with iodoform and hydrag. biniodst, the result with aristol far exceeded that with the other drugs. In all his cases the marked property of the drug in favoring cicatrization was most striking. Dr. Gaudin applied the drug either dissolved in ether or collodion, or as a dusting powder.

Dr. Hughes has found the drug of much service in all those forms of rhinitis associated with a dryness of the mucous surface, or in which there was a tendency for the secretion to undergo decomposition. He reports twenty-one cases in which he has tried the remedy, and in all the result was most satisfactory; in two cases of specific ozœna the disappearance of the fœtor and the healing were very rapid.

Dr. Löwenstein has treated four cases of ozœna with the drug and reports most favorably on its effect in one case of specific ozœna. In addition to insufflation of the drug in powder, he painted the ulcerated part with a solution in collodion flexile. He speaks very favorably of the property the drug has of forming a covering over the diseased surface.

Professor Neisser has made experiments on the action of aristol on bacteria, and also used it therapeutically in thirteen cases of lupus exulcerans, in which he found it of great service. In lichen rubra, soft chancre and gonorrrhœa the results were not satisfactory.

Dr. Schirren has treated ten cases of psoriasis of various forms, vulgaris, guttata nummularis, with aristol, and reports all the cases as cured after, at longest, twelve days' treatment. He applied the drug at 10 per cent. ointment in lanolin or vaseline, or with zinc starch. In none of the cases was any ill by-effect observed.

Dr. Schuster records a case of syphilis of the naso-pharynx, in which he had a very good result, after the use of aristol in powder. The case had been under treatment for some months without any benefit, potassium iodide being administered internally and mercury by inunction. Dr. Schuster reports also a case of psoriasis in which he used aristol with very rapid and good results. He applied a 10 per cent. solution of the drug in collodion flexile.

Dr. Seifert has made an extended trial of the new drug in the syphilis clinic at Wurzburg and reports very favorably on its use. He has used it in ulcers of the leg, lupus, psoriasis, moist condylomota and suppurating gummata, and in all cases in which it was applied the good result was most marked. Dr. Seifert states that he did not find any iodine in the urine when aristol was administered internally.

Dr. v. Swiecicki has made a trial of the drug in gynæcological practice. He has used aristol in twenty cases, and in all the drug has had had a beneficial action. The cases reported comprise endometritis, hyperplasia of the cervix, parametritis, eczema vulvæ, and, after operation for fissure of the cervix, applied as a dusting powder.—*Medical Chronicle*, *July*, *1890*.

ABADIE (CH.). "Pathogenesis and new treatment of sympathetic ophthalmitis."—*Semaine Médicale*, July 23d, 1890, p. 254.

The researches of Leber and Deutschmann have conclusively proved that sympathetic opthalmitis is a disease, caused by microbes, of an infectious origin. It is more rational to admit that the disease is transmitted by continuity of tissue from the infected optic nerve to the chiasma, and thence to the optic nerve of the sound eye, rather than by means of the ciliary nerves and nervous centres. It can easily be explained, from our knowledge of the evolution of microbes and their action on the tissues, how, first of all, the deeper parts of the exciting eye are disorganized, then the optic nerve is attacked, and the chiasma, with the optic nerve of the sound eye, and lastly, the process takes place in the sympathizing eye, which is also totally destroyed. Enucleation, the former remedy against the disease, was proposed and carried out as a result of clinical observation; no real explanation was offered as to its efficacy.

If enucleation is practiced at an early period of the disease of the exciting eye, the optic nerve is severed in the midst of sound tissues, and propagation of the inflammatory process is at once checked ; clinical observers were always unanimous in approving of enucleation before sympathetic disorders commenced. At a later stage, when sympathetic disorders have shown themselves, there was a difference of opinion ; some advised enucleation, but others forbade it. But the exciting eye contains the original focus of disease ; in it the microbes multiply with great rapidity, for they are situated in the midst of a wounded tissue, which has not much vital energy, and they are soon able to attack and destroy the healthy parts of the eye. Hence, by enucleating the exciting eye, a great point is gained in taking away the original focus of disease in which the pathogenic microbes are developing ; but still there is sympathetic disease in the second eye, and it is necessary to cure this. Several different circumstances may present themselves: If the inflammation of the sympathizing eye has only just begun, the microbes no longer reinforced by others coming from the wounded eye, soon disappear in the presence of the healthy tissues, owing to the destructive action of the phagocytes or normal cells of the tissue (?), and the eye recovers ; inunction with mercurial ointment will help the patient to get better.

If, on the other hand, the enucleation is performed at a late period, the sympathizing eye will already have its tissues profoundly altered it, and the microbes, continuing to multiply, will completely disorganize it unless some treatment directed against them be added to the enucleation.

It is also certain that, in case of an eye which has been attacked by sympathetic disease, and the latter has been arrested by enucleation or by other means, no operation should be performed on this eye for a long time, lest the morbid process, not quite exhausted, be awakened to further activity, and the tissues are still too feeble to resist the renewed attacks of the microbes.

The following conclusions may be drawn from researches and bacteriological experiments : First, enucleation, performed before the optic nerve is affected, is a certain remedy against sympathetic disease ; if it be done later, when the second eye is attacked, it may still be useful, especially if the patient be subjected to a mercurial course, and, in any case, can never aggravate the evil, since it always suppresses the primary source of the evil.

To-day, however, we can do much more than this, and can lay down the following rules :

If an eye be severely wounded, so that, from a functional and æsthetic point of view, it seems to be irredeemably lost, it may be enucleated at once, but usually, by means of the following treatment, we are convinced that this will not be necessary. In the first place, the wound should be thoroughly cleansed with antiseptics in order to avoid infection ; but if the latter occurs, the wound should be cauterized with a fine galvano-cautery, in its whole extent and minute recesses, while the patient is under chloroform. If, in spite of these procedures, the ophthalmia progresses, the idea occurred to me to inject into the eye which was destined to be removed one or two drops at the most of a 1:1,000 solution of corrosive sublimate. These injections cause, for several hours, a very lively irritation, but the reaction gradually subsides, and the condition of the two eyes improves considerably.

The most remarkable case I have ever seen which forms the point of departure for this new method of treatment, may be here related. A woman, 60 years of age, fell down a staircase and burst her left eye. When seen, several hours after the accident, an enormous rupture, nearly two centimetres long, of the sclerotic was observed, nearly at equal distance between the cornea and the equator of the eye. Through this rupture the lens had been expelled, and the ciliary body projected beyond its margins. The wound was carefully washed with a 1:2,000 solution of sublimate, and an antiseptic dressing applied. The patient went about her usual work, and came regularly to be dressed. All went well for three weeks, when sympathetic ophthalmia occurred, and progressed so rapidly that the patient could hardly see to get about. Before proceeding to enucleate the exciting eye, which was nearly cicatrized, I resolved to make an attempt to save it, and cauterized with the galvano-cautery the almost insignificant prolapse of the ciliatory body, and then by means of a Pravaz syringe injected two drops of a 1:500 solution of sublimate into the wounded eye. The reaction was exceedingly severe and the pain great. On the morrow there was found intense pericorneal redness, and complete opalescence of the posterior surface of the cornea with the membrane of Descemet. But gradually, on the following days, this reaction subsided, and the cornea became transparent again; at the same time, vision in the sympathizing eye rapidly ameliorated. Briefly, in a fortnight, not only was the vision of the sympathizing eye become nearly normal, but the wounded eye itself, which had been condemned to be enucleated, had improved so much that the patient could count fingers held at a distance of several meters, and could have seen her way about. At the present time, four months after the treatment was begun, the cure is complete, and the patient can read with the wounded eye when a convex lens of fifteen dioptrics is placed before it.

A second instructive case is the following: A child, fourteen years old, was slightly wounded in one eye, and was attacked by sympathetic ophthalmitis as a result, which had caused the vision in both eyes to be very bad and almost the same on each side. I injected into the wounded eye two drops of a 1:1,000 solution of sublimate; the reaction was much less than in the former case, and great amelioration of vision took place in both eyes.

Lastly, this third case offers a greater interest than either of the preceding ones. I had been obliged, before the new therapeutic measures which I have just related, to enucleate the right eye of a patient attacked with sympathetic disease. In spite of the enucleation, and the employment of mercurial inunctions, the sympathetic ophthalmitis continued to progress in the left eye, and vision became gradually less acute. I then decided to inject into this eye—the only one that remained—one drop of a 1 : 1,000 solution of sublimate. The disease was arrested.

Relying on these facts, I think that the therapeutic treatment of sympathetic ophthalmia has entered upon a new phase. Enucleation should be reserved for those very exceptional cases where extensive injury destroys all hope of preserving the eye. In the case of serious injury, if, in spite of antiseptic measures, sympathetic ophthalmia has occurred, it is preferable, before enucleating the eye, to cauterize the wound with a galvano-cautery, and then to inject one drop of a 1 : 1,000 solution of corrosive sublimate. Lastly, if, in spite of everything, whether intervention has been too late, or enucleation itself has been powerless, there is only one eye left, it is best to wait until the virulence of the microbes which are invading this eye has been exhausted, before performing any operation (iridectomy, or extraction of lens), with a view to improve the vision ; and this object will be the more easily obtained by making an injection into this eye of 1 : 1,000 solution of corrosive sublimate.—*Medical Chronicle, August, 1890.*

"DUPLEX INSTANTER" CLINICAL THERMOMETER.—Under this name Mr. James J. Hicks, of Hatton Garden, introduces to the profession an ingenious clinical thermometer. The bulb consists of two tubes running parallel to each other but joined throughout by a piece of solid glass. This not only gives extra sensitiveness, but greater strength. The section drawing illustrates the tubes as they would appear if cut through the centre, the darker portions showing the mercury and the lighter the binding of solid glass. There is no aperture for dirt or matter of any kind to pass or lodge between the tubes, and they are well adapted for inserting in the mouth or in any other part of the body, as they have a broader and flatter surface than ordinary thermometers. This is a great point, as there is often a difficulty in taking a sick person's temperature when inserting the thermometer under the tongue and trying to get him to keep his mouth tightly closed, owing to the uncomfortable nature of the bulb, which frequently causes the thermometer to get broken. This is especially the case with children. For the same reason the flattened surface is an advantage when the thermometer is applied to the axilla, as the absence of any sharp point or edge renders it less annoying to the patient.

This thermometer is made in a neat and compact form, occupies no more room in the pocket than any ordinary clinical thermometer, and is supplied with or without a magnifying tube. It is a distinct improvement.

v. MERING (J.) and MINKOWSKI (O.). "Diabetes mellitus after extirpation of the pancreas."—*Archiv für experimentelle Pathologie.* Bd. XXVI., Heft 5 and 6, and *Centralblatt für klinische Medicin*, June 7, 1890.

Previous experiments on extirpation of the pancreas had been attended with practically negative results.

The authors of the paper performed extirpation of the pancreas in dogs. This most difficult operation succeeded, when strict antiseptic precautions were used.

After complete extirpation of the pancreas the dogs became diabetic. There was produced, not a temporary glycosuria, but a genuine permanent diabetes, corresponding to the severest form of this disease in man. The sugar excreted was grape sugar, and the dogs were abnormally voracious and had a great thirst. If allowed to drink as much water as they desired, polyuria occurred. In spite of the large amount of food which they took, they soon became thin and feeble. Sooner or later the urine contained, besides grape sugar, a great quantity of acetone, aceto-acetic acid, and oxybutyric acid. The amount of sugar in the blood was considerably increased. The glycogen of the organs disappeared early.

The diabetes continued until death. Mostly the animals died from complications in connection with bad healing of the wounds.

The latter was due to the diminished resistance of the diabetic animals to the evil results of wound infection. None of the animals lived longer than four weeks. Of those cases in which the wounds healed well, some died of inanition, some from lung complications. A striking post-mortem appearance in those animals was the marked fatty degeneration of the liver.

The diabetes appearing after extirpation of the pancreas is not the result of injury of adjacent parts, such as the solar plexus. The best proof of this, is the result of partial pancreas extirpation. If portions of the pancreas of various size were removed, but still a small piece of the gland were left, no diabetes was produced ; even when the excretory duct was twice ligatured, and the pancreas dissected away from the duodenum.

The occurrence of diabetes is thus a direct result of the absence of the pancreas. The absence of the pancreatic juice in the intestinal canal is not the cause. Diabetes occurred after starvation for a long period, when the intestinal canal was completely empty.

A disturbance in the intermediate metabolism must take place after the extirpation of the pancreas. These experiments point to a special and hitherto unknown function of the pancreas, by which the destruction of sugar (taken with the food or formed in the body) is brought about. This is a specific function of the pancreas. If an animal, made diabetic by extirpation of the pancreas, be fed with a definite quantity of grape sugar (after the sugar excretion has become almost constant under a uniform flesh diet), then the whole of the grape sugar is excreted in the urine. This would not take place if still another organ besides the pancreas could bring about the destruction of the sugar.

In addition to diabetes, as a result of pancreas extirpation, there occurred also a deficient absorption of fat and albuminous bodies in the intestinal canal.—*The Medical Chronicle, July, 1890.*

